# Tick infestation on medium–large-sized mammalian hosts: are all equally suitable to *Ixodes ricinus* adults?

**DOI:** 10.1186/s13071-021-04775-6

**Published:** 2021-05-13

**Authors:** Atle Mysterud, Christian Hügli, Hildegunn Viljugrein

**Affiliations:** 1 Centre for Ecological and Evolutionary Synthesis (CEES), Department of Biosciences, University of Oslo, Blindern, P.O. Box 1066, 0316 Oslo, Norway; 2 Norwegian Veterinary Institute, Sentrum, P.O. Box 750, 0106 Oslo, Norway

**Keywords:** Hosts, Ticks, Host competence, Tick abundance, Tick prevalence

## Abstract

**Background:**

In Europe, the generalist tick, *Ixodes ricinus*, is the main vector of several tick-borne pathogens causing diseases in humans and livestock. Understanding how different species of hosts limit the tick population is crucial for management. In general, larger ectoparasites are expected to select hosts with larger body size. Consistent with this, larval and nymphal *I. ricinus* can feed on a wide range of different-sized vertebrates, while the adult female stage is expected to rely on a medium–large-sized host for reproduction. However, we still have a limited understanding of whether medium-sized hosts other than roe deer can serve as hosts to adult ticks, and other factors than size may also affect host selection.

**Methods:**

To increase our understanding of the suitability of the different species of medium-sized hosts for adult ticks, we sampled mainly roadkill mammals from within the questing season of ticks. We counted life stages of ticks on roe deer (*Capreolus capreolus*) (*n* = 29), red fox (*Vulpes vulpes*) (*n* = 6), badger (*Meles meles*) (*n* = 14) and red squirrel (*Sciurus vulgaris*) (*n* = 17) from spatially overlapping populations in Norway, and analysed variation between species across different body parts with a mixed-effects negative binomial model (with and without zero-inflation).

**Results:**

Red squirrel hosted a high density of larval and nymphal *I. ricinus*, but only one individual had adult female ticks. Roe deer hosted by far the largest number of adult ticks. Badgers had very few ticks, possibly due to their thick skin. Red foxes had intermediate numbers, but a high proportion of subcutaneous, dead ticks (69.3%), suggesting they are not very suitable hosts. Body mass predicted the presence of adult *I. ricinus* ticks. However, species was a better predictor than body mass for number of ticks, suggesting there was species variation in host suitability beyond body mass per se.

**Conclusions:**

Our study provides evidence that roe deer are indeed the main suitable reproduction host to adult *I. ricinus* ticks, and are likely a key to host limitation of the tick population in this northern ecosystem.

**Graphic abstract:**

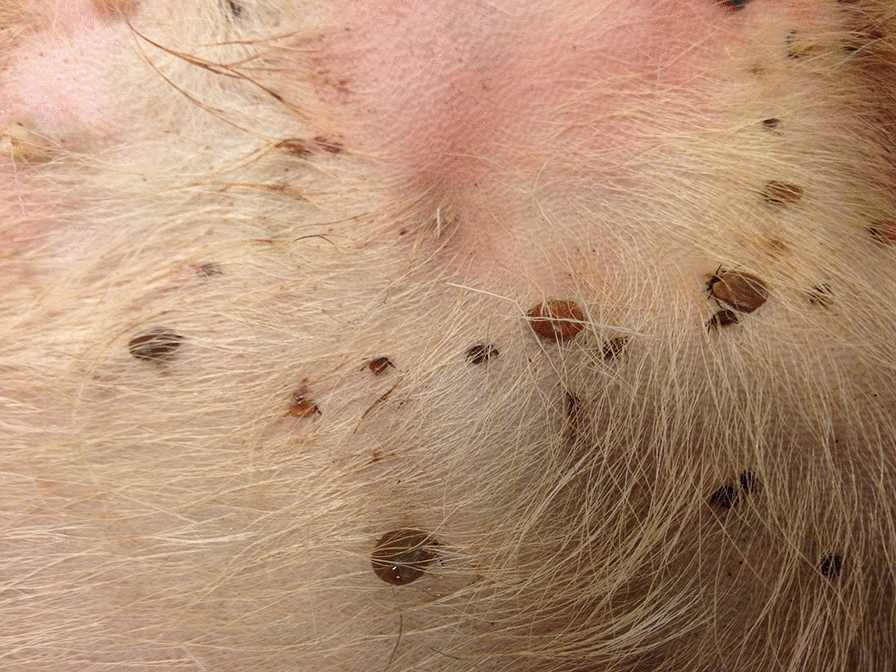

## Background

Tick-borne diseases are emerging across northern latitudes in both Europe [1] and North America [2], and understanding the role of the host community for limiting tick-borne diseases is a key issue for management. In Europe, the generalist tick *Ixodes ricinus* is the main vector of the pathogens causing Lyme borreliosis [3] and other pathogens that are of medical importance [4]. *I. ricinus* is a host generalist, feeding on a wide range of vertebrates [5]. *I. ricinus* require a new blood meal as larvae, nymph and adult, but there is an ontogenetic shift in host selection. Larval ticks are mainly found on small vertebrates, while nymphal ticks are found on a wide range of different-sized vertebrates. Adult ticks require a blood meal from a medium–large-sized vertebrate host to reproduce. Hosts for the adult stage are regarded as particularly critical for limitation of ticks and tick-borne diseases [6, 7].

In North America, there are claims that the limited role of deer for limitation of tick-borne diseases is due to other medium-sized mammalian hosts being available as reproduction hosts to adult ticks [8]. In Europe, the mammal community consists of fewer species in the medium-sized segment compared to North America, and deer species seem to play a role in limiting tick-borne diseases in Europe [9–11]. In northern ecosystems, host alternatives to deer for ticks are mainly red foxes (*Vulpes vulpes*) and badgers (*Meles meles*), while hedgehogs (*Erinaceus europaeus*) are rare compared to in continental Europe. Further, it is uncertain whether the red squirrel (*Sciurus vulgaris*) is sufficiently large to be a suitable host to adult ticks, as previous sampling included only a small number (*n* = 6) of squirrels [12].

Parasite and host size in general are correlated, but finding quantitative rules for scaling of parasite to host body size has proven difficult [13, 14]. There may be several other factors—morphological (body shape, thickness of skin), behavioural (grooming) or physiological (immune system)—affecting host suitability to ticks [15, 16]. We still have a limited understanding of the role of different-sized hosts for the different life stages of ticks. Specifically, there is little documentation of what constitutes a sufficiently large host to adult ticks, and whether all those medium-sized mammals are equally suitable as hosts. We aim to answer this based on tick counts of different body parts of roe deer (*Capreolus capreolus*), badger, red fox and red squirrel from sympatric populations in south Norway.

## Methods

### Study area

The main study area is the municipality of Vestby in Viken county (59° 36′ 18.40″ N, 10° 45′ 8.39″ E), in the south-eastern part of Norway and close to the Oslofjord. All the species included here are common in the area (see Mysterud et al. [17] for further details on the study area).

### Data collection and processing

Data on roadkill roe deer (*n* = 29; 15 males, 14 females), red fox (*n* = 6; 4 males, 2 females), badger (*n* = 14; 7 males, 7 females) and one red squirrel were collected mainly during May–July 2014–2016. Additionally, red squirrels were sampled in August 2016 with a special permit obtained from the Norwegian Environment Agency to hunt outside the hunting season (total *n* = 17; 7 males, 10 females). Age class (juvenile, yearling or adult) was known for 71.4% of the animals included [18]. For roe deer, red fox and badger, we skinned the animals and retrieved skin from relevant body parts for tick collection [19, 20], which is recommended for larger hosts [21]. Body mass was measured for complete individuals to the nearest 100 g for roe deer, badger and red fox, and to the nearest 1 g for red squirrel. We measured the area (cm^2^) of the skin samples, apart from ear and head, and these latter parts were not used in comparison of tick density. When required, we used an electric razor to remove hair. This was done in steps of increasingly shorter hair to detect both engorged and recently attached stages. Squirrels were not skinned, and our tick counts did not differentiate between body parts. All ticks were identified as species and life stage (larvae, nymph, male adult, female adult) under stereomicroscope.

### Statistical analysis

We used negative binomial models with the package glmmTMB in R version 4.0.3 to analyse variation in tick infestation across species, body parts, sex or age groups. We mainly analysed tick abundance (number of ticks), but also analysed density of ticks (by adding log area of skin as an offset term) to determine whether differences in abundance were due to variation in surface area. To enable comparison of body parts from species with different body configurations, we merged parts into five categories (back, belly, head, leg, neck). We used a random term for individual ID to account for repeated measures of the same individual. Squirrels were not included in this comparison, as we only did a full body count rather than on specific parts. We did a separate negative binomial model comparing across species (pooled body parts) and focusing on abundance of adult ticks. We used the Akaike information criterion corrected for small sample sizes (AICc) for comparing fit of different models. We tested whether zero-inflated models led to a better model fit [22], that is, a model separating the process of excess zero counts (absence of ticks) from the number of ticks (negative binomial part). Due to relatively few data compared to the complexity of model structure, neither interaction effect nor zero-inflation were included in the model selection for the mixed-effects negative binomial model.

## Results

**Fig. 1:**
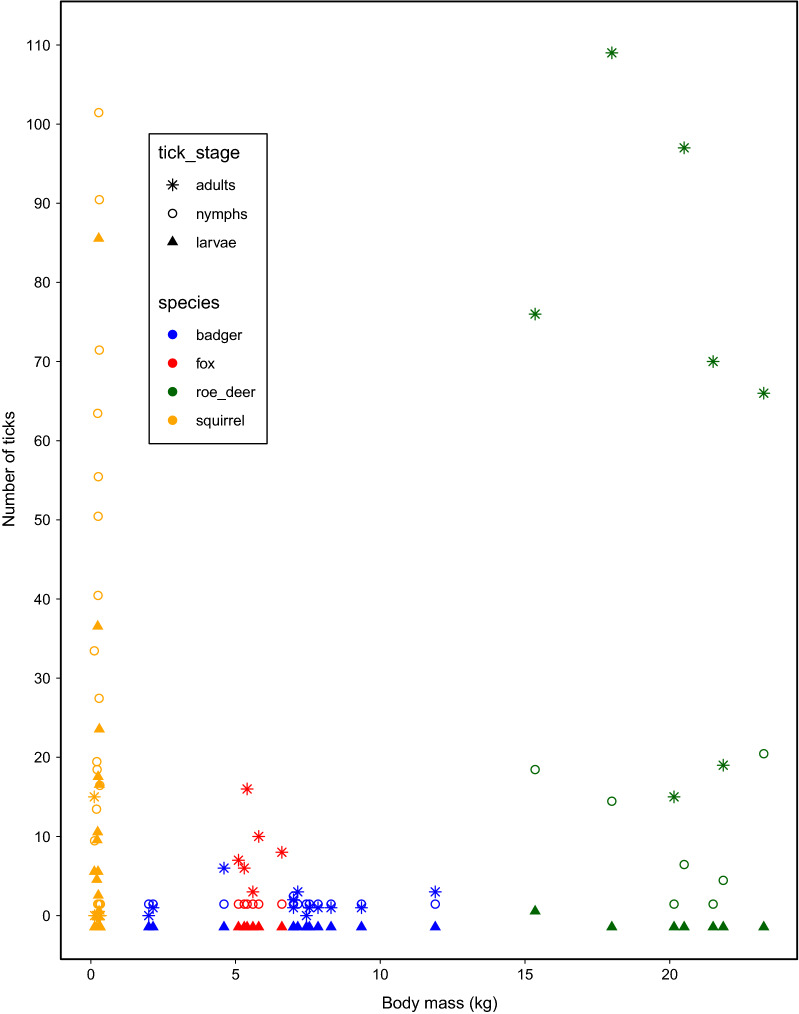
The relationship between abundance of larval, nymphal and adult life stages of *Ixodes ricinus* and body mass across four mammalian species in Norway

All ticks were *I. ricinus*. Very high tick abundance was detected on red squirrels in the form of both larvae and nymphs, but only a few adult ticks were recorded on a single individual (Table 1). The number of ticks was similar for male and female squirrels (Z = −0.393, *p* = 0.694). Tick abundance was higher on roe deer than on red fox, which in turn was higher than badger (Table 2), and tick abundance on adult hosts was higher than on younger animals (yearlings and cubs). In general, legs had lower tick abundance than other body parts, and belly had lower abundance than neck. The model fit decreased more by removing species (ΔAICc = 54.6) or body part (ΔAICc = 50.7), compared to age category (ΔAICc = 1.6). Results were broadly similar whether counts of ticks or density of ticks was used, although there was no longer a significant difference in tick abundance between fox and badger when using density of ticks. Adding sex to the model did not improve fit (∆AICc = 2.0). On the six red foxes, we found 69.3% (*n* = 163) subcutaneous and dead ticks. Across species, prevalence and abundance of adult ticks increased with body mass (Fig. 1), and body mass was highly significant when fitted alone (Z = 5.87, *p* < 0.001). The model fit improved by adding zero-inflation as a function of body mass (∆AICc = −15.2, AICc = 200.5, Table 3). However, a model adding species (∆AICc = −23.3, AICc = 177.2) reduced the effect of body mass on tick abundance (the count part of the model, Table 3), suggesting there was species variation in host suitability beyond body mass per se.

**Table 1:** Overview of parts with ticks on different hosts with tick prevalence (proportion of individuals with ticks), mean, median and max tick abundance (number of ticks if present), proportion of life stages (as a total of all life stages) and overall density (ticks per cm^2^)

Species	Part	No.	Prevalence (%)	Mean abundance	Median abundance	Max abundance	Percentage larvae (%)	Percentage nymphs (%)	Percentage adults (%)	Overall density
Roe deer	Entire body	29	100	62	42	196	3	27	71	0.028
	Back	29	100	13	7	53	2	0	98	0.064
	Belly	58	100	7	4	30	10	1	89	0.031
	Head	29	100	10	6	33	47	0	53	NA
	Leg	116	100	6	4	25	56	10	33	0.042
	Neck	29	100	15	9	52	1	0	99	0.052
Badger	Entire body	14	71	2	1	6	0	5	95	0.001
	Back	14	7	1	1	1	0	0	100	0.016
	Belly	28	18	1	1	1	0	0	100	0.006
	Head	14	7	3	3	3	0	33	67	NA
	Leg	56	7	1	1	1	0	0	100	0.012
	Neck	14	21	2	1	5	0	0	100	0.014
Red fox	Entire body	6	100	8	7.5	16	0	0	100	0.002
	Back	6	33	3	2.5	4	0	0	100	0.008
	Belly	12	25	1	1	1	0	0	100	0.010
	Head	6	33	6	7	8	0	0	100	NA
	Leg	24	13	1	1	2	0	0	100	0.005
	Neck	6	33	2	1.5	5	0	0	100	0.014
Squirrel	Entire body	17	88	56	46	187	28	70	2	2.014

**Table 2:** Parameter estimates for number of *I. ricinus* ticks (sum of larvae, nymphs and adults) on different body parts of badger, red fox and roe deer in Norway

Parameter	Estimate	SE	z	*P*
Intercept	−2.702	0.343	−7.88	< 0.001
Back	1.014	0.255	3.98	< 0.001
Belly	0.889	0.207	4.30	< 0.001
Head	1.323	0.207	6.38	< 0.001
Neck	1.565	0.247	6.35	< 0.001
Fox	1.629	0.467	3.49	< 0.001
Roe deer	3.905	0.419	9.32	< 0.001
Young	−0.898	0.370	−2.42	0.015
Unknown age category	−0.379	0.356	−1.06	0.287

Baseline was badger for species, adults for age category and leg for body part. The model is a mixed-effects negative binomial regression. There was a random effect for individual ID to account for repeated sampling

**Table 3:** Parameter estimates for number of adult *I. ricinus* ticks on red squirrel, badger, red fox and roe deer in Norway

Parameter	Estimate	SE	z	*P*
Count part of model				
Intercept	0.984	0.533	1.84	0.065
Body mass	−0.056	0.064	−0.88	0.377
Red fox	1.454	0.380	3.83	< 0.001
Roe deer	4.301	0.883	4.87	< 0.001
Red squirrel	1.729	0.768	2.25	0.024
Zero-inflation model				
Intercept	3.191	1.135	2.81	0.005
Body mass	−1.713	0.890	−1.93	0.054

Baseline was badger for species. The model consists of a zero-inflated part (logistic regression of excess zero counts) and a count part (negative binomial regression)

## Discussion

A number of factors determine the suitability of a mammalian host to ticks and other ectoparasites [23]. Our study clearly confirms large variation among medium-sized hosts in the level of feeding ticks, and that only roe deer among the medium–large-sized mammals fed adult ticks to a large degree (Fig. 1). We still have a limited understanding of the contribution of factors other than body size to host suitability for ticks [24], such as body shape, thickness of skin, the immune system and the level of grooming [15, 16]. However, we found clear evidence of species variation in tick abundance after controlling for the effect of body mass.

All roe deer were infested with high numbers of adult ticks, as expected (Fig. 1), and confirm their important role as reproduction hosts for *I. ricinus* [6, 7]. Badgers have a low body posture searching for food in what appear typical tick habitat, and they are likely exposed to high numbers of questing ticks. Nevertheless, there were few ticks attached on badgers (Fig. 1), as also found in the Netherlands [25]. This may possibly be due to their very thick skin. Further, both red fox and badgers may (self)groom [15, 16], but tick numbers were too small to analyse whether body parts that were accessible to grooming had fewer ticks. Also, badgers have small ears, an area of the body with thin skin and shallow blood vessels, often with a high number of ticks in roe deer [19] and red deer (*Cervus elaphus*) [20]. Similarly, red fox appeared not very suitable hosts to ticks, though tick numbers were higher than for badgers. We confirmed recent findings of subcutaneous and dead ticks in red fox in the Czech Republic, Romania [26], Poland [27] and Germany [28]. As many as 69.3% of ticks were dead and in different stages of decomposition, indicative that red fox have an immune defence killing ticks. We found no *I. hexagonus*, even though we expected to find them on carnivores [25, 29]. Red squirrels had a high number of larval and nymphal ticks, but apparently they are not sufficiently large to host many adult ticks, as those were only found on a single individual and then in quite high numbers. Note that a limitation was that we could not account for seasonal or annual variation in tick abundance due to unbalanced sampling.

Population density clearly also impacts the importance of a given vertebrate host to the tick population [30]. Reliable estimates of population numbers are not available in our case, but carnivores in general occur at lower densities than deer. In the Netherlands, four species of mustelids, including badger, had a negligible contribution to the circulation of tick-borne pathogens [25]. Our study on tick abundance across species suggests this is likely also the case in a northern ecosystem of Europe.

## Conclusions

Our study suggest that roe deer is a more suitable host to adult *I. ricinus* than other medium-sized mammals like badger and red fox. We also provide confirmatory evidence that red squirrel are usually not infested with adult ticks. These insights have implications for targeting hosts in mitigation efforts.

## Data Availability

The datasets used during the current study are available from the corresponding author on reasonable request.

## Abbreviations

AICc: Akaike information criterion corrected for small sample sizes
ΔAICc: Difference in AICc between a given model and the most parsimonious model
*B. burgdorferi* s.l.: *Borrelia burgdorferi* sensu lato
*I. ricinus*: *Ixodes ricinus*
